# A Case with Hypothyrodism Following Autologous Stem Cell Transplantation

**DOI:** 10.4274/Tjh.2012.0177

**Published:** 2013-06-05

**Authors:** Berna Bozkurt Duman, Semra Paydaş, Mehtap Evran

**Affiliations:** 1 Çukurova University Medical Faculty, Department of Oncology, Adana, Turkey; 2 Çukurova University Medical Faculty, Department of Endocrinology, Adana, Turkey

## TO THE EDITOR

Hypothyroidism is the leading cause of thyroid dysfunction and can be seen in up to 40% of these patients and appropriate treatment is critical importance [1,2]. Hypothyroidism is seen most frequently in patients receiving total body irradition (TBI) containing conditioning regimens. Hypothyroidism may be seen in cases receiving chemotherapy-only conditioning regimens but less frequently [3,4,5]. Here we report a case of hypoythyroidism detected 6 months after autologous hematopoietic stem cell transplantation (HSCT) for multiple myeloma (MM). 

## CASE REPORT

A 56-year-old woman was diagnosed with Durie-Salmon stage IIIA and International Staging System stage II MM. Four cycles of VAD (Vincristine 0.4 mg for 4 days, doxorubicine 9mg/m2 for 4 days, dexamethasone 40mg for 12 days) and zoledronic acid were given. Bone marrow aspiration and biopsy was normal. Esbach was 12 g/day at the beginning and it was negative after therapy. Induction with high dose melphalan (200 mg/m2) and mobilization was performed with cyclophosphamide (4 g/m2) plus G-CSF (5-10 µg/kg/d) and HSCT was performed. Hypothyroidism was detected 6 months after transplantation. Free T3 was 0.462 pg/mL (N:T3: 2.3-4.2 pg/mL), free T4 was 0.179 ng/mL (N: 0.61-1.12 ng/mL), and TSH was 771.6 mIU/L (N: 0.34-5.6 mIU/L). She had no known prior history of thyroid dysfunction. Antimicrosomal antibody was found to be high 600.1 U/mL (normal range: less than 50) and antityroglobuline was within normal limits (2.21 mg/dL). At the beginning hypothyroidism could not be controlled and dose was increased up to 200 mcg (Table 1). TSH alpha subunit and hypophysis MR were performed. Pathologic finding was not found. She has been free of disease in follow-up for 3 years and with normal thyroid function. 

## DISCUSSION

The prevalence of posttransplant hypothyroidism is highly variable and is seen in up to 58% of the cases [1,2,3,4,5,6,7,8]. Niedzielska et al. reported on 16 patients after auto-HSCT and 30 patients after allo-HSCT; hypothyroidism was found in 5 of these patients (3 after allo-HSCT, 2 after auto-HSCT) in their series [9]. Post-transplant hypothyroidism is seen generally after a median of 1.5 to 2 years [3,4,9]. Earlier thyroid dysfunction as short as 6 months after HSCT was reported [7]. The current concept of pathogenesis immune thyroiditis after allogeneic transplantation is the transfer of a clone of donor lymphocytes with antithyroidal activity. T cells play an important role in thyroid damage and also complement-mediated injury [10]. Significant hypothyroidism can be seen after autologous transplantation receiving chemotherapy-only condiotioning regimen. High levels of autoimmune markers may suggest the immune etiology. 

## Figures and Tables

**Table 1:**
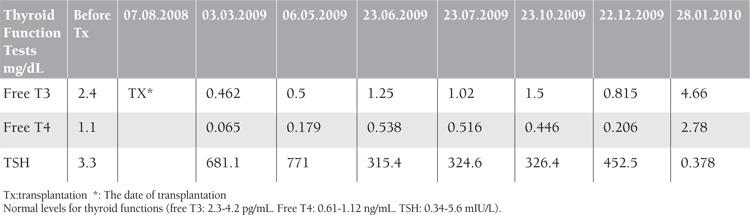
Thyroid function tests at follow up
